# Mitochondrial Toxicology of Heavy Metals and Pesticides: Transport Systems, Mitochondrial Dysfunction and Permeability Transition

**DOI:** 10.3390/ijms27146347

**Published:** 2026-07-17

**Authors:** Graziantonio Lauria, Giuseppe Genchi, Rosita Curcio

**Affiliations:** Department of Pharmacy, Health and Nutritional Sciences, University of Calabria, Via P. Bucci, 87036 Rende, CS, Italy; graziantonio.lauria@unical.it

**Keywords:** mitochondrial permeability transition pore, environmental pollutants, oxidative stress, calcium signaling, ATP synthase, ANT, MCU complex

## Abstract

Mitochondrial transport systems are essential regulators of cellular bioenergetics, calcium homeostasis, and metabolic signaling, and have emerged as critical targets of environmental toxicants. Although heavy metals and pesticides act through distinct primary mechanisms, increasing evidence indicates that they converge on a common network of mitochondrial dysfunction characterized by oxidative stress, impaired metabolite transport, calcium dyshomeostasis, and sensitization to mitochondrial permeability transition. This review provides an updated overview of the major mitochondrial transport systems involved in environmental toxicity, including the adenine nucleotide translocator (ANT), phosphate carrier (PiC), mitochondrial calcium uniporter (MCU), voltage-dependent anion channel (VDAC), and F1·Fo-ATP synthase (ATP synthase). We discuss their physiological roles, the molecular mechanisms by which heavy metals and pesticides disrupt their function, and the effects on oxidative phosphorylation, reactive oxygen species (ROS) generation, cardiolipin remodeling, and mitochondrial membrane integrity. Particular attention is devoted to the debate surrounding the molecular basis of mitochondrial permeability transition pore (mPTP) formation and to the concept that permeability transition represents the integrated outcome of cumulative mitochondrial stress rather than dysfunction of a single protein. Finally, we summarize emerging therapeutic strategies aimed at preserving mitochondrial transport function, limiting mitochondrial permeability transition, and attenuating downstream inflammatory signaling. Understanding these convergent mechanisms may facilitate the development of interventions to mitigate chronic diseases associated with environmental toxicant exposure.

## 1. Introduction

Mitochondria are central hubs of cellular metabolism and signaling, integrating bioenergetic processes with adaptive stress responses [1]. Rather than acting merely as isolated powerhouses dedicated to adenosine triphosphate (ATP) production, these multifunctional organelles coordinate metabolic fluxes, redox balance, and intracellular calcium homeostasis. Mitochondria function as critical upstream regulators of innate immunity [2], mediate retrograde signaling to the nucleus through metabolites and redox cues [1], and influence cellular fate decisions in response to environmental toxicants and stressors [3].

A distinctive feature of mitochondrial physiology is the strict compartmentalization of metabolic reactions. The inner mitochondrial membrane (IMM) is highly impermeable to most hydrophilic metabolites and ions, a property essential for maintaining the mitochondrial membrane potential (Δψ) required for oxidative phosphorylation (OXPHOS). The exchange of substrates between the cytosol and the mitochondrial matrix relies on specialized transport systems, primarily represented by the Mitochondrial Carrier Family (also known as the Solute Carrier Family 25, SLC25) [4]. This family encompasses more than fifty transport proteins responsible for the flux of nucleotides, tricarboxylic acid cycle intermediates, amino acids, cofactors, and inorganic ions across the IMM [4,5]. Because these carriers link mitochondrial bioenergetics with cytosolic pathways, alterations in their transport kinetics or structural integrity can rapidly compromise cellular homeostasis, serving as important contributors to numerous congenital and acquired disorders [4,5,6,7]. Mitochondrial transport systems act as sensitive molecular gatekeepers that translate environmental stress into bioenergetic and signaling responses [3,4]. Xenobiotics and environmental pollutants frequently target these interfaces, disrupting metabolite flows, ion dynamics, and the regulation of mitochondrial permeability transition.

Historically, environmental toxicology has investigated heavy metals and pesticides within distinct toxicological and epidemiological paradigms, with limited consideration of shared mechanisms of toxicity [3]. However, emerging evidence suggests that both classes converge on mitochondrial dysfunction as a shared mechanism of toxicity [3,8]. Heavy metals such as cadmium, mercury, lead, and arsenic frequently exert direct effects through interactions with protein thiol (-SH) groups, displacement of essential metals [9], and disruption of mitochondrial enzymes and transport systems [3]. In contrast, pesticides often initiate toxicity through class-specific primary targets, including acetylcholinesterase inhibition by organophosphates [10] and voltage-gated sodium channel modulation by pyrethroids [11], but many subsequently induce reactive oxygen species (ROS) overproduction and mitochondrial impairment [12]. The ensuing oxidative stress promotes oxidative modifications of mitochondrial proteins and electron transport chain components, thereby exacerbating mitochondrial dysfunction [13]. Despite distinct upstream initiating events, both toxicant classes converge on transport protein dysfunction [3], oxidative stress, and impaired mitochondrial Ca^2+^ homeostasis [8], a combination of signals that lowers the threshold for opening the mitochondrial permeability transition pore (mPTP) [14,15].

The opening of the mPTP serves as a critical bioenergetic checkpoint, leading to the collapse of Δψ, mitochondrial swelling, matrix depletion of crucial cofactors, and the release of pro-apoptotic factors that drive cell death [16,17]. Crucially, while the definitive molecular identity of the pore-forming unit remains a subject of intense academic debate, several IMM transport systems and selected outer mitochondrial membrane (OMM) proteins, such as the voltage-dependent anion channel (VDAC), have been proposed to regulate or associate with mPTP activity [16]. This review provides a comprehensive synthesis of how specific mitochondrial transport systems act as early molecular sensors and mediators of environmental toxicity. We analyze the functional properties of these transporters and dissect the distinct direct and indirect mechanisms by which heavy metals and major classes of pesticides disrupt their activity. Furthermore, we examine the biochemical pathways linking transporter impairment to mPTP activation, discuss the emerging downstream immunological and proteolytic consequences of pore opening, and address the paradigm of low-dose mitochondrial hormesis. Finally, we highlight promising mitochondria-targeted therapeutic strategies designed to counteract pollutant-induced transport system damage and safeguard cellular viability.

## 2. Mitochondrial Membrane Transporters and the Permeability Transition Complex: Gating and Toxicological Vulnerability

The mPTP is a regulated permeability transition phenomenon that may range from transient low-conductance ion-selective opening to sustained high-conductance permeabilization, allowing the passage of solutes up to approximately 1.5 kDa [16,18]. Persistent mPTP opening dissipates ΔΨm, promotes matrix swelling, OMM rupture, bioenergetic collapse, and the release of pro-apoptotic factors such as cytochrome c [14]. Environmental toxicants do not merely induce non-specific membrane damage but preferentially target mitochondrial transport systems involved in metabolite exchange, Ca^2+^ homeostasis, and permeability transition, including the adenine nucleotide translocator (ANT), phosphate carrier (PiC), mitochondrial calcium uniporter (MCU), VDAC, and F1·Fo-ATP synthase (ATP synthase) [8,19]. These transport systems are highly responsive to metabolic and xenobiotic stress [20] and can directly modulate the threshold for mPTP opening [19].

### 2.1. Mitochondrial Adenine Nucleotide Translocator

ANT is an abundant electrogenic carrier of the IMM that couples OXPHOS to cellular energy demand by exchanging cytosolic adenosine diphosphate (ADP) for matrix ATP [21]. This transport cycle shifts ANT between cytoplasmic-facing (c-state) and matrix-facing (m-state) conformations [22]. Beyond nucleotide exchange, ANT conformational states critically influence mPTP sensitivity. Ligands that stabilize ANT in the m-state, such as bongkrekic acid (BKA), inhibit pore opening, whereas ligands that favor the c-state, including atractyloside (AT) and carboxyatractyloside (CAT), lower the threshold for mPTP activation [18]. This conformation-dependent gating renders ANT uniquely vulnerable to environmental xenobiotics. The matrix surface of ANT contains highly conserved, critical cysteine residues (particularly Cys160 and Cys257) that are highly susceptible to oxidative modification [23]. ROS or direct thiol-reactive toxicants, such as heavy metals, may promote modification and cross-linking of these thiol groups, favoring non-selective pore formation in the presence of the matrix chaperone cyclophilin D (CypD) [18,24]. Under stress conditions, CypD is thought to interact with ANT in a conformation-dependent manner, promoting mPTP opening by lowering the Ca^2+^ threshold required for pore activation [25]. Furthermore, ANT structural integrity is fundamentally dependent on its interaction with cardiolipin, an IMM-specific phospholipid rich in unsaturated fatty acids that stabilizes the active transport dimer [26]. ROS accumulation or exposure to pro-oxidant xenobiotics induces cardiolipin peroxidation [27], decreasing its affinity for ANT and causing the protein to destabilize [28]. Oxidized cardiolipin can subsequently translocate to the OMM, where it acts as a signaling platform for the recruitment and activation of pro-apoptotic proteins such as BCL2-associated X protein and BCL2 homologous antagonist/killer, while also modulating CypD-dependent mPTP opening, thereby inducing cytochrome c release [29]. Recent genetic studies are consistent with cooperative models in which ANT and CypD jointly contribute to permeability transition under pathological conditions. Indeed, combined genetic ablation of ANT isoforms and CypD is required to almost completely suppress high-conductance permeability transition, supporting the concept that multiple molecular components cooperate in regulating pore opening rather than identifying a single definitive pore-forming unit [24]. Interestingly, specific conformational modulation of ANT can alleviate lipotoxic stress. While CAT-mediated inhibition of ANT compromises mitochondrial bioenergetics, stabilization of ANT in the m-state by BKA during palmitate overload inhibits mPTP opening, preserves Δψ, and attenuates lipotoxic cell injury, demonstrating that structural targeting of this transporter may mitigate pollutant-like cellular stress [30].

### 2.2. Mitochondrial Phosphate Carrier

The mitochondrial PiC (encoded by SLC25A3) mediates the proton-coupled influx of inorganic phosphate across the IMM, supplying the phosphate required for ATP synthesis [31]. Although genetic deletion of SLC25A3 does not abolish mPTP opening, arguing against earlier proposals that PiC constitutes the core pore-forming unit, it markedly desensitizes the pore and reduces susceptibility to Ca^2+^-induced permeability transition [32].

Thus, PiC acts as a potent regulatory rheostat. Elevated matrix phosphate enhances mPTP sensitivity by acting in concert with Ca^2+^ and promoting the formation of amorphous Ca^2+^–phosphate precipitates within the matrix [18,33]. Under sustained stress conditions, this process favors persistent mPTP opening, potentially through CypD-dependent modulation of neighboring IMM components [18]. This pathway is further amplified by inorganic polyphosphate, which promotes mPTP opening in a chain-length-dependent manner [34]. Recent studies have highlighted polyphosphate as an emerging regulator of mitochondrial bioenergetics and permeability transition [35]. Models further suggest that polyphosphate may interact with the ATP synthase c-subunit ring and polyhydroxybutyrate to influence channel conductance and selectivity [36].

### 2.3. Mitochondrial Calcium Uniporter Complex

The MCU complex is the primary high-capacity IMM channel that drives the electrogenic entry of cytosolic Ca^2+^ into the matrix [37], while accessory subunits, including MICU1, MICU2, EMRE, and MCUb, fine-tune channel activity [38]. Under physiological conditions, matrix Ca^2+^ activates pyruvate, isocitrate, and α-ketoglutarate dehydrogenases, thereby enhancing NADH production and OXPHOS [39]. However, under environmental stress, MCU becomes a critical gateway for mitochondrial damage. Xenobiotic-induced cytosolic Ca^2+^ elevations promote excessive MCU-mediated matrix Ca^2+^ accumulation [20]. This overload accelerates mitochondrial metabolism and ROS generation, thereby enhancing mPTP sensitivity and facilitating CypD-dependent regulation of IMM pore components [40]. Although CypD promotes the conformational transition underlying pore opening, this process is markedly attenuated when rapid mitochondrial Ca^2+^ uptake through the MCU complex is impaired [41].

### 2.4. Voltage-Dependent Anion Channel

VDAC is a β-barrel channel localized to the OMM, serving as the main conduit for ATP, ADP, inorganic phosphate, and respiratory substrates [42]. Depending on the transmembrane potential, VDAC gates between an open, anion-selective high-conductance state and a closed, cation-selective low-conductance state [43]. In its closed conformation, VDAC restricts ATP/ADP exchange but paradoxically exhibits enhanced permeability to Ca^2+^, a property implicated in pro-apoptotic signaling [44].

VDAC gating is highly sensitive to OMM lipid composition, which influences channel dynamics and bioenergetics [45]. Structural studies further indicate that β-barrel mobility underlies voltage-dependent channel closure [46]. In addition, metabolic cues regulate VDAC activity, as free tubulin can block the channel and reduce metabolite flux [47], whereas hexokinase II binding stabilizes VDAC and reshapes mitochondrial energetic output [48].

Because VDAC resides exclusively in the OMM, current evidence does not support its inclusion as a core structural component of the mPTP, whose pore-forming event is thought to arise within the IMM [16]. Rather, VDAC serves important regulatory and scaffolding functions that influence mitochondrial susceptibility to permeability transition. At endoplasmic reticulum (ER)-mitochondria contact sites, known as mitochondria-associated membranes (MAMs), VDAC forms a functional bridge with the ER-resident inositol 1,4,5-trisphosphate receptor (IP3R) through the cytosolic chaperone glucose-regulated protein 75 (Grp75), thereby facilitating efficient interorganellar Ca^2+^ transfer and coordinating Ca^2+^-dependent signaling between the ER and mitochondria [49].

Under basal physiological conditions, the IP3R–Grp75–VDAC complex ensures tightly regulated ER-to-mitochondria Ca^2+^ transfer required to sustain mitochondrial metabolism and OXPHOS [49]. Under toxicant exposure, dysregulated ER–mitochondria Ca^2+^ signaling promotes excessive Ca^2+^ transfer to mitochondria, overwhelming the downstream MCU complex and triggering matrix Ca^2+^ overload and mPTP opening [20,41]. Furthermore, sustained cellular stress promotes VDAC oligomerization, a process proposed to generate OMM macro-pores that facilitate the release of cytochrome c and other pro-death mitochondrial factors following permeability transition [50].

### 2.5. ATP Synthase

The ATP synthase is a multi-subunit complex enriched at the edges of mitochondrial cristae, where its dimerization contributes to IMM curvature and cristae architecture [51]. The involvement of ATP synthase in the molecular identity of the mPTP remains one of the most intensely debated issues in contemporary bioenergetics [15]. Early experimental studies proposed that, under conditions of severe stress, ATP synthase may shift from an energy-conserving enzyme into a high-conductance channel. Two major models have emerged: one implicating the c-subunit ring of the F_0_ domain as the pore-forming structure [52,53], and another proposing that the permeability pathway arises at the dimeric interface between adjacent ATP synthase monomers [54]. More recent evidence suggests that ATP synthase may cooperate with ANT in regulating permeability transition rather than acting as an isolated entity [55].

This transition is proposed to be regulated by the interaction of CypD with the oligomycin-sensitivity conferring protein subunit of ATP synthase, potentially transmitting conformational changes to the F_0_ domain, particularly under conditions in which pathological Ca^2+^ accumulation displaces matrix Mg^2+^ from regulatory sites [56,57]. However, the issue is still far from being resolved, and the “enzyme-to-pore” hypothesis should be interpreted with balanced caution [58]. Alternative models propose that ATP synthase does not directly form the high-conductance permeability pathway, but instead acts as an important upstream regulator of IMM stability and pore susceptibility [59]. Consistent with this view, mitochondria lacking the ATP synthase c-subunit have been reported to lose the canonical high-conductance permeability transition, while retaining low-conductance channels sensitive to cyclosporin A (CsA), suggesting that alternative structural pathways may contribute to permeability transition [60].

Furthermore, contradictory genetic knockout studies have yielded markedly different outcomes depending on the species and experimental systems examined, highlighting the context-dependent nature of mPTP regulation [58]. Rather than identifying a definitive pore-forming unit, these findings have revived alternative models implicating ANT-containing assemblies and other multi-protein complexes in permeability transition, while reinforcing the view that no single structural paradigm currently accounts for all experimental observations [15,16]. In this context, intact ATP synthase dimers have been proposed to function as negative regulators that preserve IMM integrity and restrain non-specific permeabilization, rather than constituting the pore itself [59]. Thus, the definitive molecular structure of the mPTP core channel remains to be elucidated, and the effects of environmental toxicants should be evaluated according to their capacity to perturb this delicate structural equilibrium and shift mitochondria toward permeability transition [15].

**Figure 1:**
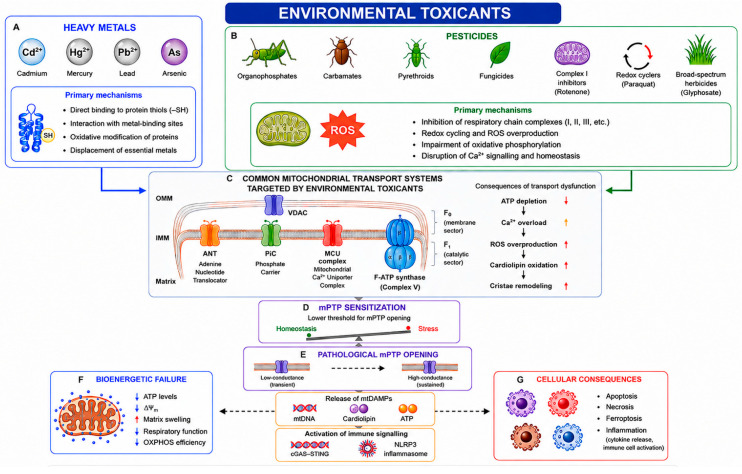
Convergent mechanisms by which environmental toxicants impair mitochondrial transport systems and promote mitochondrial permeability transition. (**A**) Heavy metals predominantly exert toxicity through direct interaction with protein thiols, metal-binding sites, and redox-sensitive mitochondrial proteins. (**B**) Pesticides primarily impair respiratory metabolism, promote oxidative stress through respiratory chain inhibition or redox cycling, and disrupt intracellular Ca^2+^ homeostasis. (**C**) Despite these different initiating mechanisms, both classes of environmental toxicants target major mitochondrial transport systems, including the adenine nucleotide translocator (ANT), phosphate carrier (PiC), mitochondrial calcium uniporter (MCU) complex, voltage-dependent anion channel (VDAC), and F1·Fo-ATP synthase (ATP synthase). Their progressive impairment compromises ATP production, promotes mitochondrial Ca^2+^ overload, enhances reactive oxygen species (ROS) generation, and induces cardiolipin oxidation and cristae remodeling. (**D**) These events progressively lower the threshold for pathological mitochondrial permeability transition pore (mPTP) opening. (**E**) Sustained high-conductance mPTP opening leads to bioenergetic failure; release of mitochondrial damage-associated molecular patterns (mtDAMPs), activation of innate immune pathways, including the cyclic GMP-AMP Synthase–Stimulator of Interferon Genes (cGAS–STING) pathway and the Nucleotide-Binding Oligomerization Domain-Like Receptor (NLR) Family Pyrin Domain-Containing 3 (NLRP3) inflammasome. Such events lead to (**F**) bioenergetic failure and (**G**) cellular consequences, including regulated cell death. The detailed molecular mechanisms governing mitochondrial transporter dysfunction, permeability transition, and adaptive mitochondrial responses are further illustrated in Figure 2 and Figure 3. (This figure was created with AI assistance using ChatGPT-5.5 and subsequently revised and scientifically validated by the authors).

The mitochondrial transport systems described above represent the principal molecular interface through which structurally diverse environmental toxicants impair mitochondrial homeostasis. Although heavy metals and pesticides act through distinct primary mechanisms, they ultimately converge on a common network of transporter dysfunction, oxidative stress, Ca^2+^ dysregulation, and progressive sensitization to mitochondrial permeability transition. An overview of these convergent pathogenic mechanisms is presented in Figure 1, providing the conceptual framework for the following sections.

**Figure 2:**
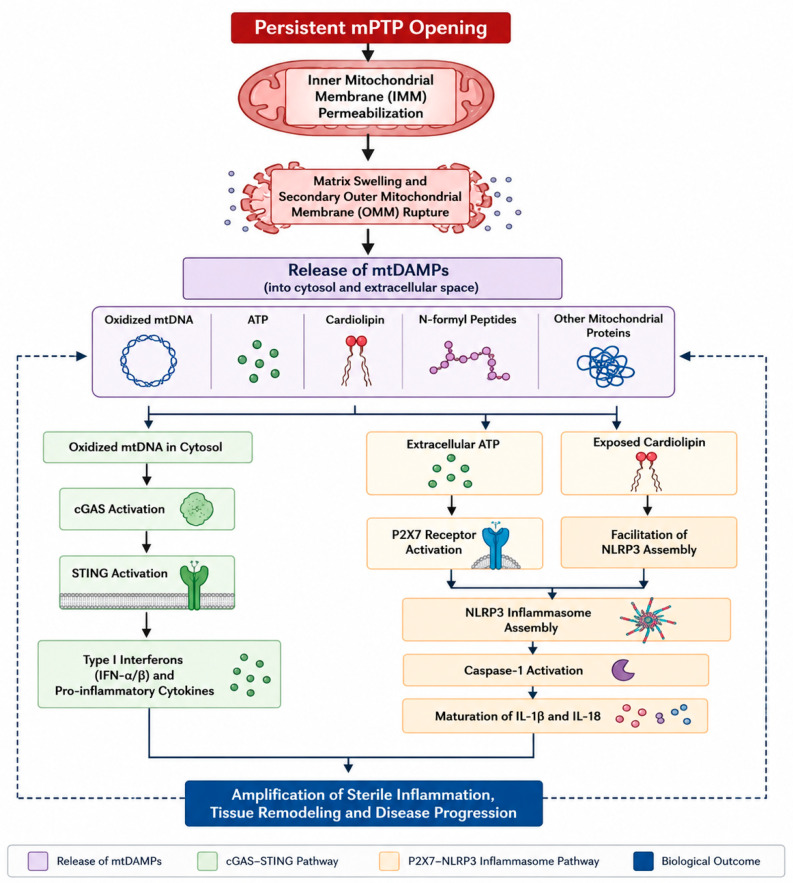
Integrative model of the downstream immunological consequences of persistent mitochondrial permeability transition. Persistent mPTP opening induces permeabilization of the inner mitochondrial membrane (IMM) and, following matrix swelling, secondary rupture of the outer mitochondrial membrane (OMM), allowing the release of mtDAMPs, including oxidized mitochondrial DNA (mtDNA), adenosine triphosphate (ATP), cardiolipin, N-formyl peptides, and other mitochondrial constituents. Cytosolic oxidized mtDNA activates the cGAS–STING pathway, whereas extracellular ATP acting through purinergic P2X7 receptors together with exposed cardiolipin promotes assembly of the NLRP3 inflammasome. The activation of these complementary innate immune pathways culminates in caspase-1 activation, maturation of interleukin 1β (IL-1β) and interleukin 18 (IL-18), amplification of sterile inflammation, tissue remodeling, and progressive disease associated with environmental toxicant-induced mitochondrial dysfunction. (This figure was created with AI assistance using ChatGPT-5.5 and subsequently revised and scientifically validated by the authors).

**Figure 3:**
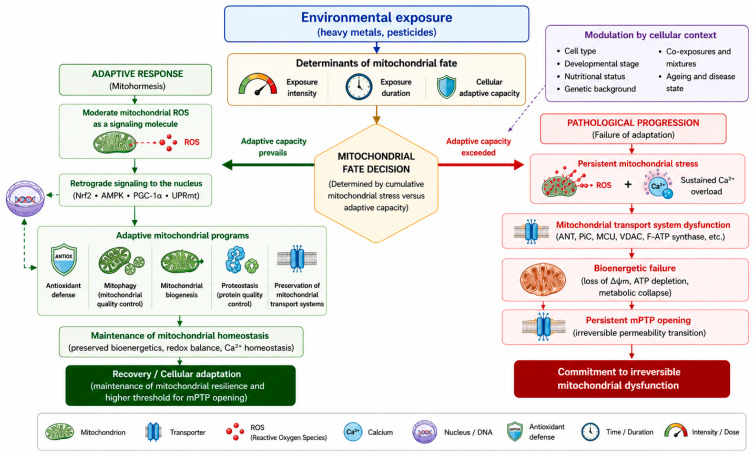
Conceptual model of mitochondrial fate determination following environmental toxicant exposure. Environmental toxicant exposure does not invariably lead to irreversible mitochondrial injury. Instead, mitochondrial fate is determined by the dynamic balance between cumulative mitochondrial stress and cellular adaptive capacity, which is influenced by exposure intensity, exposure duration, and intrinsic or extrinsic biological factors. When adaptive capacity prevails, moderate mitochondrial ROS act as signaling molecules that activate retrograde mitochondria-to-nucleus communication through nuclear factor erythroid 2-related factor 2 (Nrf2)-, AMP-activated protein kinase (AMPK)-, peroxisome proliferator-activated receptor gamma coactivator 1-alpha (PGC-1α)-, and mitochondrial unfolded protein response (UPRmt)-dependent pathways. These coordinated adaptive programs promote antioxidant defenses, mitophagy, mitochondrial biogenesis, proteostasis, and preservation of mitochondrial transport systems, thereby maintaining mitochondrial homeostasis, preserving bioenergetic function, and increasing the threshold for persistent mPTP opening. Conversely, when adaptive capacity is exceeded, sustained ROS production and persistent Ca^2+^ overload promote progressive dysfunction of mitochondrial transport systems, bioenergetic failure, and irreversible mPTP opening, ultimately committing mitochondria to irreversible dysfunction. Downstream inflammatory signaling mediated by mtDAMPs, including activation of the cGAS–STING pathway and the NLRP3 inflammasome, is summarized separately in Figure 2. (This figure was created with AI assistance using ChatGPT-5.5 and subsequently revised and scientifically validated by the authors).

## 3. Heavy Metal-Mediated Mitochondrial Toxicity and Transport Disruption

Mitochondrial dysfunction has emerged as a central mechanistic link between environmental heavy metal exposure and the development of chronic neurodegenerative disorders [12], as well as cardiovascular, renal, and metabolic diseases [8]. Rather than eliciting only generic cellular stress responses, heavy metals such as cadmium, mercury, lead, and arsenic frequently disrupt the function of mitochondrial transport systems and redox-sensitive protein domains involved in bioenergetic homeostasis [61]. Perturbation of these metabolic gatekeepers impairs ATP synthesis, alters intra-organellar redox balance, and dysregulates Ca^2+^ signaling [61], thereby lowering the activation threshold required for induction of the mPTP [14,18].

### 3.1. Common Mechanisms of Heavy Metal-Induced Mitochondrial Dysfunction

Although individual metals differ in their physicochemical properties and primary molecular targets, they ultimately converge on a limited number of pathogenic processes, including excessive ROS generation, disruption of thiol redox homeostasis, dysregulation of intracellular and mitochondrial Ca^2+^ signaling, and progressive destabilization of mitochondrial membranes [62].

Mitochondrial ROS overproduction represents one of the earliest responses to heavy metal exposure and contributes to oxidative modification of proteins, lipids, and mitochondrial DNA (mtDNA) [63]. Oxidation of reactive cysteine residues alters the activity of several mitochondrial transport proteins, whereas cardiolipin peroxidation destabilizes respiratory-chain complexes and perturbs the structural organization of the IMM [27]. These events amplify ROS production, impair OXPHOS, and progressively sensitize mitochondria to permeability transition [64].

As discussed in Section 2, mitochondrial Ca^2+^ overload is another critical determinant of permeability transition. Under physiological conditions, Ca^2+^ is transferred from the ER to mitochondria through the IP3R–Grp75–VDAC complex, which facilitates local Ca^2+^ delivery to mitochondria at ER–mitochondria contact sites [65,66]. Heavy metals potentiate this physiological pathway by inducing ER stress, disrupting intracellular Ca^2+^ homeostasis, and promoting sustained mitochondrial Ca^2+^ accumulation, thereby lowering the threshold for mPTP opening and irreversible mitochondrial injury [62].

Beyond promoting mPTP opening, persistent mitochondrial Ca^2+^ overload activates mitochondrial calpains (mitocalpains), initiating an additional proteolytic pathway that further amplifies mitochondrial dysfunction [67]. Activated mitocalpains cleave key respiratory-chain proteins, aggravating mitochondrial depolarization, ATP depletion, and oxidative stress, thereby reinforcing permeability transition and cell death [68].

Overall, these interconnected mechanisms establish the common pathogenic framework through which heavy metals impair individual mitochondrial transport systems. The following sections examine how specific metals interact with ANT, PiC, MCU, VDAC, and ATP synthase, ultimately converging on permeability transition and mitochondrial dysfunction.

### 3.2. Effects of Heavy Metals on the Adenine Nucleotide Translocator

Building on the structural and regulatory properties of ANT discussed in Section 2, heavy metals represent some of the most potent environmental modifiers of ANT function. Through direct interaction with reactive thiol residues and secondary oxidative modification of the translocator, these toxicants impair physiological ADP/ATP exchange, compromise mitochondrial bioenergetics, and increase susceptibility to permeability transition [69]. Mercury, cadmium, and other thiol-reactive metals therefore converge on ANT as a critical target linking oxidative stress to mitochondrial dysfunction [70]. Consistent with this mechanism, oxidation of ANT thiol residues promotes mPTP opening and apoptosis independently of canonical Bcl-2 signaling pathways [71].

Mercury and methylmercury, owing to their pronounced thiophilicity, are particularly effective at modifying ANT-associated thiol residues and disrupting nucleotide transport [70]. Although direct stabilization of a specific ANT conformational state by mercury species has not been conclusively demonstrated, these thiol modifications are thought to destabilize physiological ANT transport cycling and shift the translocator toward conformations associated with increased susceptibility to permeability transition, functionally resembling the effects produced by c-state-stabilizing ligands such as CAT [18]. Consistent with this interpretation, mercury-induced ANT dysfunction contributes to mitochondrial depolarization, impaired ATP production, and activation of apoptotic signaling pathways [72].

Similarly, cadmium interacts with ANT-associated thiol networks, promoting conformational alterations that facilitate mPTP opening and Δψ dissipation [73]. The central role of ANT in cadmium toxicity is further supported by the observation that pre-incubation with BKA, which stabilizes ANT in the m-state conformation, effectively abolishes Cd^2+^-induced mPTP opening and markedly attenuates downstream apoptotic responses [74]. By contrast, CsA provides only partial protection against Cd^2+^-induced mitochondrial permeabilization despite inhibiting the CypD-dependent regulation of mPTP opening [74]. These findings suggest that direct coordination of Cd^2+^ with ANT thiol residues is likely to promote conformational changes that sensitize the translocator to pore opening, thereby reducing, rather than completely bypassing, its dependence on canonical CypD-mediated regulation [73].

In highly energy-dependent tissues, including the liver, myocardium, renal cortex, and central nervous system, persistent ANT dysfunction contributes to impaired ATP export, bioenergetic collapse, and activation of both necrotic and apoptotic pathways, thereby promoting the progressive tissue injury that characterizes chronic heavy metal toxicity [72]. These mechanisms are increasingly recognized as important contributors to the development of chronic neurodegenerative, cardiovascular, and renal disorders associated with long-term environmental metal exposure [75].

### 3.3. Effects of Heavy Metals on the Phosphate Carrier

Heavy metals impair PiC function through two principal mechanisms: phosphate molecular mimicry and thiol-dependent protein modification. Among environmental toxicants, arsenic is uniquely suited to exploit the physiological substrate specificity of PiC. In its pentavalent form, arsenate (AsV) closely resembles phosphate and enters phosphate-dependent metabolic pathways through molecular mimicry [76]. Once transported into the mitochondrial matrix, arsenate can substitute for phosphate during OXPHOS, generating unstable ADP–arsenate intermediates that undergo rapid hydrolysis, thereby dissipating metabolic energy without ATP conservation [76].

In addition to substrate mimicry, thiophilic metals directly impair PiC through covalent modification of reactive protein thiols. Trivalent arsenic (AsIII), Hg^2+^, and Cd^2+^ readily interact with thiol groups on mitochondrial proteins, promoting oxidative modifications that compromise carrier activity and mitochondrial phosphate metabolism [77]. Because PiC function depends on tightly regulated conformational transitions within the IMM, oxidative and covalent modifications induced by heavy metals are expected to reduce phosphate transport efficiency, thereby exacerbating mitochondrial bioenergetic dysfunction [31]. Recent evidence further supports the concept that oxidative damage to mitochondrial carrier proteins represents a common mechanism underlying heavy metal-induced mitochondrial dysfunction [78].

Recent studies have expanded the functional significance of PiC beyond phosphate transport. The mammalian SLC25A3 carrier also mediates mitochondrial copper transport required for cytochrome c oxidase (Complex IV) biogenesis [79]. Evolutionary analyses further indicate that phosphate and copper transport are functionally integrated within the SLC25A3 family, highlighting the evolutionary conservation of these dual transport activities [80]. Consequently, heavy metal-induced PiC dysfunction may simultaneously impair phosphate utilization and mitochondrial copper homeostasis, thereby compromising cytochrome c oxidase assembly, respiratory-chain function, and mitochondrial ATP production.

### 3.4. Effects of Heavy Metals on the MCU Complex

As discussed in Section 2, mitochondrial Ca^2+^ uptake through the MCU complex is a major determinant of susceptibility to mitochondrial permeability transition. Heavy metals exploit this regulatory axis primarily by disrupting cellular Ca^2+^ homeostasis rather than by directly targeting the MCU pore. Exposure to cadmium, mercury, lead, and arsenic promotes sustained cytosolic Ca^2+^ elevations through a combination of oxidative stress, ER dysfunction, and altered plasma membrane ion transport, thereby increasing the driving force for mitochondrial Ca^2+^ uptake [61]. This convergence of oxidative stress and Ca^2+^ dysregulation is now recognized as a common mechanism underlying heavy metal-induced mitochondrial dysfunction [62].

A major mechanism involves dysregulation of ER–mitochondria communication at MAMs, which facilitates excessive Ca^2+^ transfer from intracellular stores to mitochondria [81]. Cadmium exposure has been shown to activate the IP3R–MCU signaling axis, promoting excessive Ca^2+^ influx into the mitochondrial matrix and triggering matrix Ca^2+^ overload [82]. Similar alterations in ER–mitochondria Ca^2+^ signaling have also been described for other environmental toxicants, establishing a mechanistic link between persistent ER stress, Ca^2+^ dyshomeostasis, mitochondrial dysfunction, and bioenergetic failure [12].

Persistent matrix Ca^2+^ accumulation further amplifies mitochondrial oxidative stress by impairing electron transport chain function and enhancing mitochondrial ROS production [83]. In turn, excessive ROS further aggravates mitochondrial Ca^2+^ dysregulation, establishing a self-amplifying cycle that progressively lowers the threshold for mPTP opening [61]. Beyond promoting permeability transition, sustained mitochondrial Ca^2+^ overload induces bioenergetic collapse, activates apoptotic signaling pathways, and contributes to ferroptotic cell death through ROS-dependent lipid peroxidation and mitochondrial membrane damage [84]. More broadly, these interconnected processes represent fundamental mechanisms linking mitochondrial Ca^2+^ dysregulation to oxidative stress, inflammatory signaling, and progressive tissue injury [83].

Globally, these findings identify the MCU pathway as a major amplifier of heavy metal-induced mitochondrial toxicity, linking disturbances in cellular Ca^2+^ homeostasis to mitochondrial dysfunction, permeability transition, and multiple forms of regulated cell death.

### 3.5. Effects of Heavy Metals on VDAC

Residing within the OMM, VDAC represents the primary metabolic interface regulating the flux of ions, metabolites, and adenine nucleotides between the intermembrane space and the cytosol [42,85]. Rather than directly damaging the channel pore, heavy metals primarily promote oxidative modifications of VDAC, including protein carbonylation and thiol oxidation, which may alter channel conductance and gating behavior [20,61]. Structural and biophysical studies indicate that transitions from the fully open state toward partially closed conformations reduce permeability to adenine nucleotides and other anionic metabolites, while simultaneously increasing cation permeability, including Ca^2+^ flux [44,46].

These alterations may contribute to impaired metabolic exchange across the OMM, mitochondrial energetic stress, and enhanced mitochondrial Ca^2+^ accumulation under conditions of heavy metal exposure [61]. A well-characterized example is arsenic trioxide, which has been shown to promote VDAC-dependent mitochondrial permeabilization and cytochrome c release, thereby activating downstream apoptotic signaling pathways [86]. In addition to altering channel permeability, heavy metals may disrupt protective protein interactions at the OMM. VDAC normally associates with proteins such as hexokinase II and members of the Bcl-2 family, interactions that contribute to mitochondrial integrity and cell survival. Disturbance of these complexes has been associated with increased susceptibility to mitochondrial dysfunction and apoptotic signaling [42,87].

At MAMs, VDAC1 functions as a structural component of the IP3R–Grp75–VDAC1 tethering complex that coordinates ER–mitochondria Ca^2+^ transfer. Cadmium exposure has been reported to enhance the activity of this signaling axis, thereby facilitating excessive mitochondrial Ca^2+^ loading and contributing to downstream mitochondrial dysfunction [88]. Available evidence suggests that VDAC acts as an important downstream target of heavy metal-induced oxidative and calcium-mediated stress, linking disturbances in metabolite exchange and ER–mitochondria communication to mitochondrial injury and cell death.

### 3.6. Effects of Heavy Metals on the ATP Synthase

Heavy metals impair ATP synthase function through multiple mechanisms, including modification of catalytically relevant residues, disruption of membrane lipid–protein interactions, and alteration of the supramolecular organization of the enzyme within mitochondrial cristae. Experimental evidence indicates that Cd^2+^, Hg^2+^, Pb^2+^, and other redox-active metals inhibit ATP synthase activity either through direct interaction with the enzyme or indirectly by promoting oxidative damage to mitochondrial proteins and membrane lipids [61]. These alterations contribute to impaired OXPHOS and progressive mitochondrial bioenergetic dysfunction [8].

The catalytic activity of ATP synthase is particularly vulnerable to oxidative modification because several subunits contain reactive amino acid residues susceptible to metal-induced ROS generation. Accordingly, exposure to Cd^2+^ and Hg^2+^ has been associated with reduced ATP synthase activity, decreased OXPHOS efficiency, and diminished cellular ATP production [69]. In parallel, arsenate acts as a structural mimic of phosphate, participating in futile phosphorylation reactions that generate unstable arsenylated intermediates and thereby uncouple ATP production from energy conservation [76].

Beyond its effects on catalysis, heavy metal toxicity profoundly alters the lipid microenvironment required for normal ATP synthase organization. Metal-induced ROS promotes cardiolipin peroxidation, weakening the interactions that stabilize respiratory-chain supercomplexes and ATP synthase oligomers within cristae membranes [27]. Because cardiolipin is essential for maintaining ATP synthase dimerization and cristae architecture, disruption of this phospholipid environment compromises IMM organization and reduces mitochondrial bioenergetic efficiency [51,89].

Heavy metal exposure also promotes mitochondrial Ca^2+^ overload and oxidative stress, two major determinants of mitochondrial permeability transition [61]. These conditions further destabilize ATP synthase organization and its surrounding lipid environment, thereby increasing mitochondrial susceptibility to permeability transition [83]. However, whether heavy metals directly induce conversion of ATP synthase into a pore-forming structure remains unresolved. Current evidence supports the concept that alterations of ATP synthase should be interpreted within the broader and still unresolved debate concerning the molecular identity of the mPTP, as discussed in Section 2 [15,16].

## 4. Pesticide-Induced Mitochondrial Dysfunction and Transport Impairment

Whereas heavy metals predominantly exert toxicity through direct interactions with protein thiols, metal-binding sites, and other redox-sensitive domains, pesticides more commonly induce mitochondrial dysfunction indirectly by inhibiting respiratory metabolism, enhancing oxidative stress, disrupting intracellular Ca^2+^ homeostasis, and impairing mitochondrial bioenergetics [3]. Despite these mechanistic differences, both toxicant classes ultimately compromise mitochondrial transport systems, promote ROS generation, and increase susceptibility to permeability transition, thereby converging on common pathways of mitochondrial dysfunction and cell death [8].

Pesticides comprise a chemically diverse group of compounds capable of affecting mitochondrial function through multiple complementary mechanisms. Although direct interactions with mitochondrial proteins have been reported for selected pesticides, mitochondrial injury most frequently results from secondary alterations in OXPHOS, redox homeostasis, Ca^2+^ signaling, and mitochondrial membrane integrity [90]. These disturbances progressively impair the activity of mitochondrial carriers and channels located in both the OMM and IMM, thereby amplifying bioenergetic dysfunction and increasing mitochondrial vulnerability to permeability transition [12].

Depending on their chemical class and primary molecular targets, pesticides may induce oxidative and nitrosative stress, alter membrane lipid organization, perturb intracellular Ca^2+^ homeostasis, or interfere directly with components of the OXPHOS machinery [13]. Because mitochondrial transport systems are tightly integrated with respiratory metabolism, these primary toxic effects are rapidly propagated to mitochondrial carriers and transport complexes, resulting in impaired metabolite exchange, altered ion homeostasis, and progressive mitochondrial dysfunction [20].

The following sections summarize how the major pesticide classes affect specific mitochondrial transport systems and membrane-associated regulatory pathways involved in bioenergetics, Ca^2+^ handling, permeability transition, and cell death.

### 4.1. Organophosphates, Carbamates, and Synthetic Pyrethroids: Oxidative and Structural Disruption

Organophosphates (e.g., chlorpyrifos) and carbamates are among the pesticide classes most extensively associated with mitochondrial dysfunction. Beyond their classical cholinesterase-inhibiting activity, these compounds promote mitochondrial ROS generation, lipid peroxidation, and loss of Δψ, thereby impairing OXPHOS and cellular bioenergetics [90]. Mitochondrial dysfunction induced by these pesticides is increasingly recognized as a major contributor to their chronic toxic effects beyond cholinergic toxicity [3].

The resulting oxidative and nitrosative stress promotes post-translational modifications of mitochondrial proteins, including carbonylation, thiol oxidation, and S-glutathionylation [91]. These oxidative modifications compromise the activity of redox-sensitive mitochondrial transport systems, particularly ANT and PiC, whose transport cycles critically depend on the integrity of reactive thiol residues [3]. More broadly, oxidative modification of mitochondrial carrier proteins has emerged as a common mechanism linking pesticide exposure to mitochondrial transport dysfunction [12].

In parallel, pesticide-induced cardiolipin peroxidation disrupts the lipid microenvironment required for the structural organization of IMM proteins, including mitochondrial carriers and OXPHOS assemblies [27]. Because cardiolipin is essential for the stability of ANT-containing assemblies, respiratory supercomplexes, and ATP synthase oligomers, its oxidation further impairs mitochondrial transport efficiency and OXPHOS [92].

Synthetic pyrethroids, particularly cypermethrin, additionally perturb intracellular Ca^2+^ homeostasis. Experimental evidence indicates that cypermethrin induces ER stress and activates the IP3R1–GRP75–VDAC1 signaling axis, thereby enhancing Ca^2+^ transfer from the ER to mitochondria [93]. The resulting increase in ER-to-mitochondria Ca^2+^ flux promotes excessive MCU-mediated matrix Ca^2+^ accumulation, leading to mitochondrial swelling, enhanced ROS production, and increased susceptibility to permeability transition [12]. These findings further support the concept that disruption of mitochondrial Ca^2+^ homeostasis represents a common downstream mechanism through which structurally diverse pesticides converge on mitochondrial dysfunction.

Overall, organophosphates, carbamates, and synthetic pyrethroids impair mitochondrial function through complementary mechanisms involving oxidative protein damage, cardiolipin remodeling, and Ca^2+^ dyshomeostasis. These interconnected events progressively compromise mitochondrial transport systems, promote bioenergetic failure, and sensitize mitochondria to permeability transition and regulated cell death.

### 4.2. Fungicides: Energetic Impairment and Secondary Transport Dysfunction

Agricultural fungicides disrupt mitochondrial function primarily through inhibition of respiratory metabolism and secondary induction of oxidative stress. Among these compounds, the dithiocarbamate fungicide maneb has been extensively investigated because of its marked mitochondrial toxicity, particularly in neuronal models, where it impairs respiratory chain activity and enhances mitochondrial ROS production [94]. Mitochondrial dysfunction is now considered a major contributor to the neurotoxic effects associated with chronic exposure to this class of pesticides [3].

Respiratory chain inhibition compromises generation of the proton motive force required to sustain OXPHOS and ATP synthesis. As a consequence, mitochondrial transport systems that depend on Δψ, including ANT, PiC, and ATP synthase, undergo progressive functional impairment, resulting in defective ADP/ATP exchange, reduced phosphate transport, and diminished ATP production [12]. These bioenergetic alterations further destabilize mitochondrial homeostasis and increase susceptibility to permeability transition [27].

Particularly severe mitochondrial dysfunction has been reported during combined exposure to maneb and paraquat, a pesticide mixture widely employed as an experimental model of environmentally associated Parkinsonian neurodegeneration [95]. The synergistic interaction between these toxicants markedly enhances oxidative stress, impairs mitochondrial respiration, and accelerates dopaminergic neurodegeneration, supporting the concept that mitochondrial dysfunction represents a central mechanism underlying their combined neurotoxicity [96]. Consistent with this interpretation, experimental studies have shown that co-exposure to maneb and paraquat amplifies mitochondrial oxidative damage and promotes activation of apoptotic cell death pathways [97].

### 4.3. Specific Complex Inhibitors and Broad-Spectrum Herbicides: Rotenone, Paraquat, and Glyphosate

Among pesticides that impair mitochondrial function, rotenone, paraquat, and glyphosate-based herbicides represent three mechanistically distinct paradigms of mitochondrial toxicity. Although they target different primary molecular processes, all ultimately converge on excessive ROS production, bioenergetic failure, mitochondrial Ca^2+^ dysregulation, and increased susceptibility to permeability transition, thereby providing complementary models for understanding environmentally induced mitochondrial injury [3,8].

Rotenone is a naturally occurring botanical insecticide and one of the best-characterized inhibitors of mitochondrial Complex I (NADH:ubiquinone oxidoreductase). By blocking electron transfer from iron–sulfur centers to ubiquinone, rotenone promotes electron leakage toward molecular oxygen, primarily at the flavin mononucleotide site, thereby markedly increasing mitochondrial superoxide production [98]. This mechanism has been extensively exploited in experimental models of Parkinson’s disease because chronic Complex I inhibition faithfully reproduces several pathological features of dopaminergic neurodegeneration, including selective degeneration of nigrostriatal neurons and progressive motor impairment [99]. Persistent oxidative stress results in ATP depletion, mitochondrial depolarization, and progressive impairment of mitochondrial transport systems, thereby facilitating Ca^2+^ overload and prolonged mPTP opening [100]. Beyond inducing apoptosis through cytochrome c release, rotenone-mediated mitochondrial dysfunction also promotes release of mitochondrial damage-associated molecular patterns (mtDAMPs), including oxidized mtDNA, thereby activating the cyclic GMP-AMP Synthase–Stimulator of Interferon Genes (cGAS–STING) pathway and the Nucleotide-Binding Oligomerization Domain-Like Receptor (NLR) Family Pyrin Domain-Containing 3 (NLRP3) inflammasome, mechanisms increasingly implicated in chronic neuroinflammation [40,101,102].

Unlike rotenone, paraquat does not primarily inhibit electron transport, but acts as a potent redox cycler. Following one-electron reduction by mitochondrial and cytosolic oxidoreductases, paraquat is continuously reoxidized by molecular oxygen, generating sustained superoxide production while regenerating the parent compound and establishing a self-perpetuating oxidative cycle [103]. Mitochondria are among the principal intracellular sites of this process, leading to progressive oxidation of respiratory complexes, cardiolipin, and mtDNA together with loss of Δψ and impaired ATP synthesis [104]. This vicious cycle progressively amplifies mitochondrial ROS production, lowers the Ca^2+^ threshold required for permeability transition, and culminates in mitochondrial swelling, outer membrane permeabilization, and apoptotic cell death [105]. Consistent with these mechanisms, paraquat exposure is strongly associated with persistent mitochondrial dysfunction and sustained inflammatory responses in experimental models of Parkinsonian neurodegeneration and pulmonary injury [105].

Glyphosate-based herbicides exhibit a broader and comparatively less selective pattern of mitochondrial toxicity. Glyphosate itself shows limited direct affinity for respiratory complexes; however, commercial formulations, particularly those containing surfactants, impair OXPHOS far more efficiently by disrupting electron transport, dissipating Δψ, and reducing ATP synthesis [106]. Experimental studies further demonstrate inhibition of respiratory Complexes II and III together with increased ROS production and mitochondrial swelling, effects that are generally more pronounced after exposure to formulated products than to glyphosate alone [107,108]. Secondary impairment of ATP synthase activity, together with disruption of mitochondrial membrane integrity, further compromises OXPHOS and increases mitochondrial susceptibility to permeability transition [106]. Nevertheless, the severity of these mitochondrial alterations remains strongly dependent on the specific formulation, surfactant composition, dose, and exposure paradigm employed, highlighting the importance of distinguishing glyphosate from glyphosate-based herbicides when interpreting toxicological data [107].

Taken together, rotenone, paraquat, and glyphosate-based herbicides exemplify three distinct routes by which pesticides converge on a common downstream pathogenic cascade. Whether initiated by direct inhibition of respiratory complexes, persistent redox cycling, or generalized impairment of OXPHOS, these toxicants ultimately disrupt mitochondrial transport systems, promote oxidative stress and Ca^2+^ dysregulation, facilitate pathological mPTP opening, and activate inflammatory and regulated cell death pathways. This convergence reinforces the concept that mitochondrial transport dysfunction represents a central mechanistic hub linking chemically diverse pesticides to environmental mitochondrial toxicity.

### 4.4. Vulnerability of the ATP Synthase to Pesticide Actions

The ATP synthase represents an important downstream target of several pesticide classes through both direct enzymatic inhibition and secondary disruption of mitochondrial bioenergetics [3]. Among the best-characterized direct inhibitors are organotin compounds, historically employed as agricultural pesticides and biocides, which bind the membrane-embedded F_0_ domain of ATP synthase and interfere with proton translocation across the IMM [109]. By dissipating the proton motive force, these compounds markedly reduce mitochondrial ATP synthesis and compromise OXPHOS [110].

In contrast, most contemporary pesticides affect ATP synthase indirectly through mitochondrial oxidative stress rather than direct enzyme inhibition. Redox-active compounds such as paraquat promote sustained ROS generation, leading to oxidative modification of ATP synthase subunits together with peroxidation of cardiolipin, the phospholipid required for ATP synthase dimerization and cristae organization [103]. Concurrent mitochondrial Ca^2+^ overload and cardiolipin remodeling further destabilize ATP synthase oligomers, impair cristae architecture, and reduce OXPHOS efficiency, increasing mitochondrial susceptibility to permeability transition [111].

As discussed in Section 2, whether these structural alterations directly convert ATP synthase into the pore-forming unit of the mPTP remains unresolved. Current evidence indicates that pesticide-induced oxidative and structural remodeling of ATP synthase should be interpreted within the broader context of mPTP regulation rather than as proof of a direct enzyme-to-pore transition [16]. Alternative models continue to support regulatory roles for ANT-containing assemblies and other IMM components, emphasizing that the molecular identity of the high-conductance pore remains an active area of investigation [112].

Overall, the available data indicate that ATP synthase should be considered as a bioenergetic target that is highly vulnerable to pesticide-induced oxidative stress. Whether impaired by direct enzymatic inhibition or by secondary alterations in ROS homeostasis, cardiolipin integrity, and Ca^2+^ signaling, dysfunction of ATP synthase contributes to mitochondrial bioenergetic collapse and lowers the threshold for permeability transition without necessarily identifying the enzyme as the sole structural component of the mPTP.

The following table (Table 1) provides a comparative summary of xenobiotic actions on mitochondrial transport systems.

## 5. Integrative Mechanisms Linking Mitochondrial Transport Dysfunction to Permeability Transition

### 5.1. Convergence of Mitochondrial Stress Signals

As discussed in the preceding sections, heavy metals and pesticides ultimately converge on a limited number of interconnected pathogenic mechanisms. Heavy metals predominantly modify thiol-containing proteins and other redox-sensitive mitochondrial components [8], whereas pesticides primarily impair respiratory metabolism and intracellular Ca^2+^ homeostasis [3]. Despite these distinct initiating events, both toxicant classes progressively disrupt mitochondrial transport systems, thereby promoting oxidative stress, bioenergetic failure, and increased susceptibility to mitochondrial permeability transition [121].

Rather than acting as isolated molecular events, dysfunction of ANT immediately limits ADP/ATP exchange, whereas impaired PiC activity restricts phosphate availability for OXPHOS [7]. In parallel, excessive mitochondrial Ca^2+^ uptake through the MCU complex enhances matrix ROS production [122], while oxidative damage to cardiolipin destabilizes respiratory supercomplexes, ATP synthase oligomers, and the structural organization of the IMM [27]. These alterations are not independent but mutually reinforce one another, progressively reducing mitochondrial functional reserve and lowering the threshold required for mPTP opening [121].

Current evidence therefore indicates that pathological mPTP opening should be regarded as the integrated consequence of cumulative mitochondrial stress rather than the direct result of a single molecular lesion [16]. Sustained ROS production promotes oxidative modification of mitochondrial transport proteins and membrane phospholipids, thereby aggravating mitochondrial Ca^2+^ dysregulation [27]. Conversely, persistent matrix Ca^2+^ overload further enhances ROS generation by impairing respiratory chain function and stimulating additional oxidative damage [122]. This self-amplifying cycle culminates in mitochondrial depolarization, ATP depletion, and irreversible bioenergetic collapse [121].

Importantly, this integrated stress network also provides the mechanistic bridge to the downstream events discussed in the following sections. Once the buffering capacity of mitochondrial transport systems is exceeded, prolonged mPTP opening initiates secondary processes including activation of matrix-localized calpains and release of mtDAMPs, thereby extending mitochondrial dysfunction beyond bioenergetic failure toward sterile inflammation and progressive tissue injury [67,123].

### 5.2. From Reversible Mitochondrial Dysfunction to Irreversible Permeability Transition

Environmental toxicants rarely induce an immediate and irreversible collapse of mitochondrial function. Instead, mitochondrial injury generally develops as a dynamic continuum in which early adaptive responses progressively give way to irreversible bioenergetic failure once mitochondrial stress exceeds the organelle’s capacity for compensation [8]. During the initial phases of exposure, moderate increases in mitochondrial ROS production and matrix Ca^2+^ concentration can activate cytoprotective signaling pathways, including antioxidant defenses, mitochondrial quality-control mechanisms, and metabolic remodeling [124]. These adaptive responses transiently preserve OXPHOS and ATP production despite ongoing toxic stress [121].

The transition from reversible mitochondrial dysfunction to irreversible injury occurs when multiple stress signals accumulate simultaneously rather than sequentially. Persistent oxidative stress promotes cardiolipin peroxidation and oxidation of reactive thiol residues within mitochondrial transport proteins, thereby progressively impairing mitochondrial membrane organization and transport function [27]. At the same time, sustained matrix Ca^2+^ overload, declining ATP availability, and impaired adenine nucleotide exchange converge to facilitate CypD-dependent permeability transition [122]. Consequently, mitochondrial dysfunction can no longer be attributed to failure of individual transporters, but instead reflects collapse of the integrated transport network responsible for maintaining mitochondrial homeostasis [121].

An important concept emerging from recent studies is that mPTP opening is not an all-or-none phenomenon. Transient, low-conductance pore openings contribute to physiological regulation of mitochondrial Ca^2+^ and ROS homeostasis, whereas prolonged or repetitive high-conductance opening results in dissipation of ΔΨm, matrix swelling, OMM rupture, and interruption of OXPHOS [16]. This distinction is particularly relevant in environmental toxicology because chronic exposure to low concentrations of heavy metals or pesticides may progressively sensitize mitochondria to permeability transition long before overt cell death becomes apparent [15]. Such cumulative sensitization provides a mechanistic framework for understanding the delayed onset of mitochondrial dysfunction that characterizes many chronic neurodegenerative, cardiovascular, and metabolic disorders associated with environmental toxicant exposure [8].

Once mitochondrial quality-control mechanisms—including mitophagy and mitochondrial proteostasis—can no longer compensate for persistent damage, prolonged mPTP opening establishes a self-reinforcing cycle of respiratory chain dysfunction, excessive ROS production, ATP depletion, and progressive Ca^2+^ dysregulation [125]. This irreversible transition commits mitochondria to sustained bioenergetic collapse and creates the conditions for activation of downstream inflammatory signaling and regulated cell death pathways, which are discussed in the following section [16].

### 5.3. Biological Consequences Beyond Cell Death

Persistent mPTP opening represents a fundamental biological turning point, extending the consequences of mitochondrial dysfunction beyond bioenergetic collapse and commitment to cell death. Sustained permeabilization of the IMM progressively disrupts mitochondrial compartmentalization [126]. This enables the escape of normally sequestered mitochondrial constituents into the cytosol and extracellular environment, where they function as mtDAMPs. Among these, oxidized mtDNA, ATP, cardiolipin, N-formyl peptides, and other mitochondrial proteins act as potent endogenous danger signals capable of initiating innate immune responses [127].

Among the released mtDAMPs, oxidized mtDNA has emerged as one of the principal mediators of sterile inflammation [123]. Following its release into the cytosol, oxidized mtDNA is recognized by cGAS, leading to activation of the STING pathway and induction of type I interferons together with numerous pro-inflammatory mediators [128]. In parallel, extracellular ATP activates purinergic P2X7 receptors, whereas exposed cardiolipin facilitates assembly of the NLRP3 inflammasome at damaged mitochondria, ultimately promoting caspase-1 activation and maturation of IL-1β and IL-18 [123].

These inflammatory mechanisms are increasingly recognized as important contributors to environmental toxicology. Rotenone exposure, for example, promotes mtDNA release and activation of the cGAS–STING–NF-κB signaling axis, linking mitochondrial dysfunction to persistent neuroinflammation and NLRP3 inflammasome activation [129]. Comparable molecular mechanisms remain less extensively characterized for heavy metals; nevertheless, growing evidence indicates that chronic mitochondrial dysfunction and sustained oxidative stress similarly promote mtDAMP release and sterile inflammatory responses that contribute to progressive tissue injury rather than representing merely secondary consequences of cell death [8]. More broadly, recent advances in mitochondrial immunology indicate that release of mtDAMPs constitutes a central mechanistic link between environmental toxicant-induced mitochondrial injury and chronic inflammatory disease [126].

Overall, these findings demonstrate that persistent mPTP opening should no longer be regarded solely as the terminal event of mitochondrial dysfunction. Instead, it functions as a pivotal signaling hub that connects disruption of mitochondrial transport systems with activation of innate immunity, sterile inflammation, tissue remodeling, and progressive disease. This expanded mechanistic framework provides a strong rationale for mitochondria-directed therapeutic strategies aimed not only at preventing permeability transition itself, but also at preserving mitochondrial integrity before irreversible inflammatory signaling becomes established. The sequence of events linking persistent mPTP opening to mtDAMP release and activation of innate immune pathways is summarized in Figure 2.

## 6. Therapeutic Strategies Targeting Mitochondrial Transport Systems and Permeability Transition

The mechanistic evidence discussed throughout this review indicates that environmental heavy metals and pesticides converge on a common pathogenic cascade centered on mitochondrial transport dysfunction. Direct modification of mitochondrial carriers by heavy metals or secondary impairment of respiratory metabolism by pesticides ultimately disrupts metabolite exchange, Ca^2+^ homeostasis, and mitochondrial membrane integrity, thereby promoting pathological mPTP opening [3]. As outlined in Section 3, Section 4 and Section 5, dysfunction of ANT, PiC, MCU, VDAC, and ATP synthase progressively amplifies oxidative stress and bioenergetic failure, suggesting that these transport systems represent promising therapeutic targets rather than passive targets of toxic damage [7].

Accordingly, current therapeutic strategies are shifting from nonspecific antioxidant supplementation to mechanism-based approaches that preserve mitochondrial transport function and prevent irreversible permeability transition. This shift reflects growing recognition that indiscriminate ROS scavenging often provides limited benefit once mitochondrial transport systems have been structurally or functionally damaged [121]. Instead, pharmacological approaches capable of maintaining transporter integrity, stabilizing mitochondrial membranes, and modulating Ca^2+^ handling are expected to interrupt the toxic cascade at earlier stages of mitochondrial injury [122].

Current experimental approaches can be broadly classified into five complementary categories. These include: (i) inhibition of CypD to increase the threshold for mPTP opening; (ii) modulation of mitochondrial Ca^2+^ uptake through the MCU complex; (iii) preservation of ANT and VDAC function to maintain nucleotide exchange and ER–mitochondria communication; (iv) stabilization of cardiolipin and ATP synthase organization to preserve IMM architecture; and (v) mitochondria-targeted drug delivery systems designed to enhance intracellular selectivity while minimizing systemic toxicity [121]. Recent advances in mitochondrial nanomedicine have further expanded these strategies by enabling selective delivery of antioxidants, CypD inhibitors, and regulatory compounds directly to mitochondria, thus improving pharmacological efficacy in experimental models of mitochondrial disease and toxicant-induced injury [130].

The following sections summarize the main mitochondria-directed therapies by target, showing how preserving mitochondrial transport systems interrupts the sequence linking pollutant exposure to oxidative stress, Ca^2+^ overload, permeability transition, inflammation and regulated cell death.

### 6.1. Cyclophilin D Inhibition and Pharmacological Control of Mitochondrial Permeability Transition

Among the mitochondria-directed therapeutic strategies currently under investigation, pharmacological inhibition of CypD remains the most extensively studied approach for limiting mitochondrial permeability transition following exposure to environmental toxicants. Although mPTP molecular identity remains debated, CypD is widely recognized as its master regulator, lowering the threshold for pore opening during oxidative stress and mitochondrial Ca^2+^ overload regardless of whether the pore involves ANT, ATP synthase, or a dynamic multi-protein assembly [16]. This central regulatory role makes CypD an attractive pharmacological target for preserving mitochondrial integrity during toxicant-induced injury [15].

As already discussed, heavy metals and pesticides converge on persistent mitochondrial ROS generation, matrix Ca^2+^ accumulation, and progressive impairment of ANT, PiC, MCU, and ATP synthase. Together, these alterations promote CypD activation and enhance its interaction with IMM protein complexes, thereby facilitating permeability transition [122]. Pharmacological inhibition of CypD interrupts this final common pathway by preserving mitochondrial membrane potential, limiting matrix swelling, maintaining OXPHOS, and reducing the release of cytochrome c and other pro-death mitochondrial factors [121].

CsA was the first compound shown to inhibit permeability transition through high-affinity binding to CypD, preventing its regulatory interaction with the pore machinery [16]. Consistent with this mechanism, both pharmacological inhibition and genetic ablation of CypD markedly reduce mitochondrial susceptibility to Ca^2+^- and ROS-induced permeability transition in numerous experimental models [131]. In turn, this improves mitochondrial function and enhances resistance to oxidative injury [132].

Nevertheless, the protective efficacy of CsA appears to be context-dependent. In heavy metal toxicity, particularly following cadmium exposure, direct covalent modification of ANT thiol residues promotes permeability transition through mechanisms that are only partially dependent on CypD. Consequently, although CsA significantly delays mitochondrial dysfunction, it often fails to completely prevent permeability transition when transport proteins themselves have undergone irreversible structural modification [73]. Similar observations have been reported using isolated mitochondria exposed to Cd^2+^, in which BKA-mediated stabilization of ANT provided greater protection than CypD inhibition alone [74]. These findings indicate that CypD inhibitors are likely to be most effective when combined with strategies aimed at preserving the structural integrity of mitochondrial transport systems rather than as stand-alone interventions [71].

To overcome the calcineurin-dependent immunosuppressive effects associated with CsA and improve mitochondrial selectivity, several second-generation cyclophilin inhibitors—including NIM811, alisporivir (Debio-025), and sanglifehrin derivatives—have been developed. These compounds retain high affinity for CypD while lacking significant immunosuppressive activity, and have shown encouraging results in experimental models by preserving mitochondrial bioenergetics, limiting permeability transition, and reducing tissue injury [133]. Although clinical translation remains at an early stage, these agents illustrate the ongoing shift from nonspecific cytoprotection toward mechanism-based therapies targeting the molecular regulators of mitochondrial permeability transition.

### 6.2. Modulation of Mitochondrial Calcium Transport

Because mitochondrial Ca^2+^ overload is a major trigger of heavy metal- and pesticide-induced mitochondrial dysfunction and permeability transition, modulation of mitochondrial Ca^2+^ transport has emerged as a promising therapeutic strategy that preserves OXPHOS, limits secondary ROS generation, and increases the threshold for pathological mPTP opening [134]. This strategy directly addresses one of the central pathogenic mechanisms discussed throughout this review, namely the progressive dysregulation of mitochondrial Ca^2+^ transport following toxicant-induced disruption of ER–mitochondria communication and mitochondrial transport systems [121].

Among the available approaches, inhibition of mitochondrial Ca^2+^ uptake through the MCU complex has received the greatest experimental attention. Ruthenium Red and its more selective derivative Ru360 reduce matrix Ca^2+^ accumulation by limiting MCU-mediated influx, thereby preserving Δψ and decreasing susceptibility to permeability transition [114]. Consistent with this mechanism, experimental MCU inhibition also attenuates ferroptotic neuronal death by preventing mitochondrial Ca^2+^ overload and secondary oxidative injury [84]. Additional preclinical studies further support its neuroprotective potential in mitochondrial disorders [135].

The therapeutic relevance of MCU modulation is highlighted by environmental toxicants exploiting this pathway during disease development. Specifically, cadmium stimulates excessive mitochondrial Ca^2+^ uptake through activation of the IP3R–MCU axis [82], while paraquat alters mitochondrial dynamics and Ca^2+^ regulation in Parkinsonian neurodegeneration models [136]. These findings provide a direct mechanistic rationale for targeting mitochondrial Ca^2+^ transport as an upstream intervention capable of interrupting the pathogenic cascade before irreversible permeability transition occurs.

Mitochondrial Ca^2+^ homeostasis depends not only on regulated Ca^2+^ influx but also on efficient Ca^2+^ extrusion. Recent structural studies have improved our understanding of the mitochondrial Na^+^/Ca^2+^ exchanger (NCLX) and its role in mitochondrial Ca^2+^ efflux [137]. Functional studies further demonstrate that NCLX prevents pathological matrix Ca^2+^ accumulation [138]. Future therapeutic strategies may therefore combine partial inhibition of MCU-mediated Ca^2+^ uptake with preservation of physiological NCLX-dependent Ca^2+^ extrusion, maintaining mitochondrial Ca^2+^ signaling without compromising normal bioenergetic function [139,140].

### 6.3. Mitochondria-Targeted Antioxidants and Redox Protection of Mitochondrial Transport Systems

Oxidative stress is a major mechanism by which heavy metals and pesticides impair mitochondrial transport proteins. Oxidative modification of cysteine residues, cardiolipin peroxidation, and disruption of respiratory-chain organization compromise the function of ANT, PiC, VDAC, and ATP synthase, lowering the threshold for permeability transition [27]. Consequently, current antioxidant strategies aim to preserve the mitochondrial redox environment rather than non-specifically scavenge ROS throughout the cell [141].

Among mitochondria-targeted antioxidants, mitoquinone (MitoQ) is the best-characterized example. Conjugation to the lipophilic triphenylphosphonium cation enables its selective accumulation within mitochondria, where it limits cardiolipin peroxidation, preserves mitochondrial membrane potential, and maintains the structural integrity of respiratory complexes and mitochondrial carrier proteins [142]. Consistent with these effects, MitoQ improves mitochondrial bioenergetics and attenuates oxidative injury in multiple experimental models [143].

Recently, particular attention has been focused on SS-31 (elamipretide), a tetrapeptide capable of penetrating mitochondria that selectively interacts with cardiolipin. By stabilizing this phospholipid, SS-31 preserves cristae architecture, supports respiratory supercomplex assembly, and indirectly maintains the structural integrity of ATP synthase and SLC25 family transporters during oxidative stress [144]. More recent studies further demonstrate the preservation of mitochondrial ultrastructure, along with increased respiratory efficiency and greater ATP production following treatment with SS-31 [145,146].

Additional mitochondria-targeted antioxidants, including SkQ1 and MitoTEMPO, similarly reduce mitochondrial ROS accumulation and protect membrane lipids and transport proteins from oxidative damage [142]. Beyond direct ROS scavenging, maintenance of the mitochondrial thiol redox state represents an additional strategy for preserving transporter activity. N-acetylcysteine replenishes intracellular glutathione, thereby protecting reactive cysteine residues within mitochondrial carrier proteins from oxidative modification and reducing susceptibility to permeability transition [147].

Finally, pharmacological support of mitochondrial bioenergetics may complement redox-directed therapies. Coenzyme Q10, idebenone, and related compounds improve electron transport chain function and ATP synthesis under conditions of oxidative stress [148], enhancing mitochondrial resilience even when transporter function is only partially preserved [149]. Available evidence indicates that preserving the mitochondrial redox environment is not simply an antioxidant strategy but also a means of maintaining the structural and functional integrity of mitochondrial transport systems, thereby delaying permeability transition and limiting downstream inflammatory and cell death pathways. 

### 6.4. Targeting VDAC and Mitochondrial Membrane Stability

Although VDAC1 is no longer considered a structural component of the mitochondrial permeability transition pore, it remains a central regulator of mitochondrial metabolism, metabolite exchange, ER–mitochondria communication, and apoptosis [42]. Consequently, preservation of VDAC function has emerged as a complementary therapeutic strategy aimed at maintaining mitochondrial homeostasis during toxicant-induced injury rather than directly inhibiting permeability transition [150].

Environmental toxicants alter VDAC function through multiple mechanisms, including oxidative modification of channel-associated proteins, disruption of membrane lipid organization, and dysregulation of ER–mitochondria Ca^2+^ signaling. These alterations impair metabolite exchange across the OMM, facilitate OMM permeabilization, and amplify mitochondrial dysfunction independently of the molecular mechanism responsible for mPTP formation [85]. Experimental evidence further indicates that paraquat exploits VDAC1-associated redox activity to sustain oxidative stress [120], whereas arsenic and other toxicants perturb VDAC-dependent signaling through oxidative modification and altered ER–mitochondria communication [86,151].

These observations have stimulated the development of pharmacological strategies aimed at stabilizing VDAC conformation rather than directly blocking channel activity. Small-molecule inhibitors of VDAC1 oligomerization, including VBIT-4 and VBIT-12, preserve OMM integrity, reduce cytochrome c release, and attenuate apoptotic signaling in several experimental models [152]. More recent studies further support VDAC stabilization as a strategy for maintaining mitochondrial homeostasis under conditions of persistent oxidative stress [153].

Complementary approaches have focused on disrupting pathological interactions between VDAC and Bcl-2 family proteins. Cell-permeable peptides targeting these protein–protein interactions reduce mitochondrial outer membrane permeabilization while preserving physiological metabolite exchange [154]. Although these interventions do not directly regulate permeability transition, they limit the propagation of mitochondrial injury downstream of transporter dysfunction. They may therefore serve as valuable adjuncts to therapies targeting mitochondrial Ca^2+^ transport and CypD-dependent regulation of the mPTP.

### 6.5. Nanomedicine and Emerging Mitochondria-Directed Therapeutic Strategies

Despite substantial progress in identifying pharmacological modulators of mitochondrial transport systems, successful clinical translation remains limited by inadequate mitochondrial delivery, poor tissue selectivity, and dose-limiting systemic adverse effects. These limitations have driven the development of nanotechnology-based delivery platforms that selectively transport therapeutic molecules to mitochondria, improving intracellular accumulation while minimizing off-target toxicity [130].

Mitochondria-targeted nanoparticles have been engineered to deliver antioxidants, CypD inhibitors, nucleic acids, peptides, and other bioactive molecules directly to the mitochondrial compartment. By increasing local drug concentration within mitochondria, these delivery systems enhance the efficacy of compounds designed to preserve mitochondrial transport function, maintain bioenergetic homeostasis, and delay permeability transition [155].

Recent advances in mitochondrial biology also indicate that therapeutic intervention should extend beyond preventing mPTP opening itself. Persistent permeability transition promotes the release of mtDAMPs, including oxidized mtDNA, ATP, cardiolipin, and N-formyl peptides. These molecules activate innate immune pathways centered on cGAS–STING and the NLRP3 inflammasome [123]. Experimental evidence from rotenone-induced neurotoxicity further demonstrates that activation of the cGAS–STING–NF-κB axis represents an important downstream consequence of mitochondrial dysfunction and contributes to chronic neuroinflammation [129].

Accordingly, future therapeutic strategies will likely integrate preservation of mitochondrial transport systems with selective modulation of downstream inflammatory pathways. Pharmacological inhibition of cGAS–STING signaling or NLRP3 inflammasome activation may complement therapies targeting mitochondrial dysfunction. Such approaches could limit sterile inflammation even when permeability transition cannot be completely prevented. Such combination approaches may be particularly beneficial during chronic exposure to heavy metals and pesticides, where persistent mitochondrial dysfunction and sustained innate immune activation drive progressive tissue injury.

Taken together, these advances support a mechanism-based therapeutic paradigm. Preserving mitochondrial transport systems, maintaining Ca^2+^ and redox homeostasis, modulating permeability transition, and controlling mtDAMP-mediated inflammatory signaling should be viewed as complementary rather than independent therapeutic objectives. This integrated strategy reflects the central concept of this review. Although environmental toxicants initially target different mitochondrial components, they ultimately converge on a common network of transport dysfunction, permeability transition, and chronic inflammatory signaling. This shared pathogenic network provides multiple opportunities for therapeutic intervention.

## 7. Conclusions, Emerging Concepts, and Future Perspectives

Throughout this review, we have shown that dysfunction of ANT, PiC, the MCU complex, VDAC, and ATP synthase progressively impairs ATP production, mitochondrial Ca^2+^ homeostasis, redox balance, and inner membrane integrity. These alterations lower the threshold for mitochondrial permeability transition. Rather than representing independent toxicological events, these alterations reinforce one another through interconnected positive feedback loops. As a result, the mPTP emerges as a central integrative hub linking transporter dysfunction to bioenergetic collapse, regulated cell death, and inflammatory signaling [121]. Although the precise molecular architecture of the pore remains actively debated, current evidence indicates that mitochondrial transport systems cooperate functionally in determining susceptibility to permeability transition rather than acting as isolated pathogenic targets [15,16].

An equally important concept emerging from recent research is that mitochondrial responses to environmental toxicants are highly dynamic and depend on both the magnitude and duration of exposure. Sustained or high-dose exposure promotes persistent mPTP opening, bioenergetic failure, mtDAMP release, and activation of inflammatory pathways. By contrast, transient sublethal mitochondrial stress may trigger adaptive responses collectively referred to as mitohormesis [156]. Recent studies have identified mitohormesis as a coordinated adaptive program in which moderate mitochondrial ROS production acts as a signaling intermediate. This response promotes retrograde communication between mitochondria and the nucleus and activates Nrf2-, AMPK-, PGC-1α-, and UPRmt-dependent pathways that preserve mitochondrial function and improve cellular resilience [124]. Rather than representing a simple antioxidant response, these integrated mechanisms promote mitochondrial biogenesis, mitophagy, proteostasis, metabolic remodeling, and maintenance of mitochondrial transport function, ultimately delaying the transition toward irreversible permeability transition (Figure 3).

This adaptive plasticity emphasizes that mitochondrial transport systems should no longer be viewed as passive toxicological targets but rather as dynamic molecular sensors capable of integrating metabolic stress, Ca^2+^ signaling, redox homeostasis, and mitochondrial quality control. Consequently, the biological outcome of environmental exposure is determined not solely by the chemical nature of the toxicant but also by the capacity of mitochondrial adaptive mechanisms to maintain transporter function and preserve mitochondrial homeostasis before irreversible permeability transition occurs [121].

Although remarkable progress has been made in elucidating the molecular basis of mitochondrial toxicology, several important questions remain unresolved. The structural organization of the mPTP under physiological and pathological conditions continues to be actively debated, and further investigation is required to clarify how ANT, ATP synthase, PiC, and other mitochondrial transport proteins cooperate during permeability transition. Equally important is the need to move beyond conventional acute high-dose experimental paradigms toward chronic low-dose exposure models that better reproduce real-world environmental conditions. Future studies should incorporate exposome-based approaches, pollutant mixtures, and longitudinal experimental designs. In parallel, integrating spatial proteomics, lipidomics, metabolomics, and single-cell multi-omics will facilitate the identification of early biomarkers of mitochondrial transporter dysfunction and clarify the molecular events preceding irreversible permeability transition [141].

Recognition that mitochondrial transport dysfunction represents a common mechanistic denominator linking chemically diverse environmental toxicants provides a strong rationale for mechanism-based therapeutic intervention. Future strategies will likely combine preservation of mitochondrial transport systems, modulation of Ca^2+^ and redox homeostasis, inhibition of persistent mPTP opening, attenuation of mtDAMP-mediated inflammatory signaling, and advanced mitochondria-targeted drug delivery platforms.

Overall, the evidence reviewed here supports a shift from the traditional view of mitochondria as passive toxicological targets toward a dynamic model in which mitochondrial transport systems determine whether environmental stress culminates in successful adaptation or irreversible permeability transition. This conceptual framework may guide the development of mechanism-based biomarkers and precision mitochondria-directed therapies for environmentally associated diseases.

## Figures and Tables

**Table 1:** Molecular mechanisms by which heavy metals and pesticides impair mitochondrial transport systems and promote mitochondrial dysfunction.

Xenobiotic Class (Representative Compounds)	Primary Mitochondrial Target	Principal Molecular Mechanism	Major Mitochondrial Consequences	Core References
Heavy metals (Hg^2+^, MeHg, Cd^2+^)	ANT (SLC25A4/A5)	Covalent modification of reactive ANT cysteinyl residues promotes conformational alterations that impair ADP/ATP exchange and increase susceptibility to permeability transition.	Impaired ADP/ATP transport, mitochondrial depolarization, bioenergetic failure, increased susceptibility to mPTP opening, and apoptosis.	[23,72,74,113]
Heavy metals/metalloids (AsV)	PiC (SLC25A3)	Molecular mimicry of inorganic phosphate enables PiC-mediated arsenate uptake and futil arsenylation reactions during OXPHOS.	Formation of unstable ADP–arsenate, defective phosphate utilization, impaired ATP synthesis, and mitochondrial energetic failure.	[31,76]
Heavy metals (Cd^2+^, Pb^2+^, Hg^2+^)	MCU complex (MCU–EMRE–MICU1/2)	Oxidative stress and ER dysfunction disrupt cellular Ca^2+^ homeostasis, promoting excessive MCU-mediated mitochondrial Ca^2+^ uptake.	Mitochondrial Ca^2+^ overload, oxidative stress, bioenergetic dysfunction, enhanced susceptibility to mPTP opening, and apoptosis.	[37,61,82,114]
Heavy metals/metalloids (Cd^2+^, As_2_O_3_)	VDAC1 and MAMs	Redox-mediated alteration of VDAC function together with disruption of IP3R–Grp75–VDAC signaling at ER–mitochondria contact sites.	Enhanced ER-to-mitochondria Ca^2+^ transfer, mitochondrial Ca^2+^ overload, oxidative stress, permeability transition, apoptosis, and autophagy.	[44,86,88]
Organotins (e.g., tributyltin)	ATP synthase (Fo sector)	Direct inhibition of proton translocation through the Fo sector dissipates the proton motive force and suppresses OXPHOS.	Reduced ATP synthesis, bioenergetic collapse, mitochondrial dysfunction, and increased susceptibility to permeability transition.	[109,110]
Organo-Phosphate Pesticides (e.g., chlorpyrifos)	Inner mitochondrial carrier systems (including ANT and PiC)	ROS-mediated oxidative Modification of mitochondrial membranes and carrier proteins associated with lipid peroxidation and protein carbonylation.	Impaired metabolite transport, defective OXPHOS, bioenergetic dysfunction, and enhanced susceptibility to permeability transition.	[115,116]
Synthetic pyrethroids (e.g., cypermethrin)	VDAC1 and MAMs	ER stress-dependent activation of the IP3R1–GRP75–VDAC1 signaling axis enhances ER-to-mitochondria Ca^2+^ transfer.	Mitochondrial Ca^2+^ overload, oxidative stress, bioenergetic dysfunction, and apoptosis.	[93]
Fungicides (e.g., maneb)	Energy-dependent mitochondrial carrier systems (including ANT)	Respiratory dysfunction and oxidative stress dissipate the proton motive force required for mitochondrial carrier activity and ATP synthesis.	Impaired nucleotide transport, reduced ATP production, mitochondrial energetic collapse, and enhanced susceptibility to regulated cell death.	[117]
Botanical pesticide (rotenone)	Respiratory complex I (secondary effects on MCU)	Complex I inhibition promotes mitochondrial ROS generation, ATP depletion, and secondary mitochondrial Ca^2+^ overload.	Oxidative stress, impaired OXPHOS, mitochondrial Ca^2+^ dysregulation, permeability transition, inflammatory signaling, and apoptosis.	[118,119]
Herbicide (paraquat)	VDAC-associated mitochondrial redox system	VDAC-associated NADH-dependent redox cycling generates sustained superoxide production, amplifying mitochondrial oxidative stress.	Lipid peroxidation, mitochondrial dysfunction, bioenergetic failure, inflammatory signaling, and regulated cell death.	[120]

## Data Availability

No new data were created or analyzed in this study. Data sharing not applicable.

## Abbreviations

The following abbreviations are used in this manuscript:

ADP	Adenosine Diphosphate
AMPK	AMP-Activated Protein Kinase
ANT	Adenine Nucleotide Translocator
AT	Atractyloside
ATP	Adenosine Triphosphate
BCL-2	B-Cell Lymphoma 2
BKA	Bongkrekic Acid
CAT	Carboxyatractyloside
cGAS–STING	cyclic GMP-AMP Synthase–Stimulator of Interferon Genes
CsA	Cyclosporine A
CypD	Cyclophilin D
ΔΨm	Mitochondrial Membrane Potential
EMRE	Essential MCU Regulator
ER	Endoplasmic Reticulum
F1·Fo-ATP synthase	ATP Synthase
Grp75	Glucose-Regulated Chaperone Protein 75
IMM	Inner Mitochondrial Membrane
IL-1β	Interleukin 1β
IL-18	Interleukin 18
IP3R	Inositol-1,4,5-Triphosphate Receptor
MAMs	Mitochondria-Associated Membranes
MCU	Mitochondrial Calcium Uniporter
MICU1	Mitochondrial Calcium Uptake 1
MICU2	Mitochondrial Calcium Uptake 2
MitoQ	Mitoquinone Mesylate
mPTP	Mitochondrial Permeability Transition Pore
mtDAMPs	Mitochondrial Damage-Associated Molecular Patterns
mtDNA	Mitochondrial DNA
NADH	Nicotinamide Adenine Dinucleotide
NCLX	Na^+^/Ca^2+^ Exchanger
NF-κB	Nuclear Factor-κB
NLRP3	Nucleotide-Binding Oligomerization Domain-Like Receptor [NLR] Family Pyrin Domain-Containing 3
Nrf2	Nuclear Factor Erythroid 2-Related Factor 2
OMM	Outer Mitochondrial Membrane
OXPHOS	Oxidative Phosphorylation
PiC	Phosphate Carrier
PGC-1α	Peroxisome Proliferator-Activated Receptor Gamma Coactivator 1-Alpha
ROS	Reactive Oxygen Species
SkQ1	10-(6′-plastoquinonyl)decyltriphenylphosphonium
SLC25	Solute Carrier Family 25
SS-31	Elamipretide
MitoTEMPO	2,2,6,6-Tetramethylpiperidine 1-oxyl
UPTmt	Mitochondrial Unfolded Protein Response
VBIT-12	N-[[1-(1-naphthalenylmethyl)-4-(phenylamino)-4-piperidinyl]carbonyl]-glycine
VBIT-4	N-(4-chlorophenyl)-4-hydroxy-3-[4-[4-(trifluoromethoxy)phenyl]piperazin-1-yl] butanamide
VDAC	Voltage-Dependent Anion Channel
